# Publisher Correction: Gaze, visual, myoelectric, and inertial data of grasps for intelligent prosthetics

**DOI:** 10.1038/s41597-020-0404-z

**Published:** 2020-02-17

**Authors:** Matteo Cognolato, Arjan Gijsberts, Valentina Gregori, Gianluca Saetta, Katia Giacomino, Anne-Gabrielle Mittaz Hager, Andrea Gigli, Diego Faccio, Cesare Tiengo, Franco Bassetto, Barbara Caputo, Peter Brugger, Manfredo Atzori, Henning Müller

**Affiliations:** 1 Information Systems Institute, University of Applied Sciences Western Switzerland (HES-SO Valais), Sierre, Switzerland; 2 Rehabilitation Engineering Laboratory, Department of Health Sciences and Technology, ETH Zurich, Zurich, Switzerland; 3 Istituto Italiano di Tecnologia, Genoa, Italy; 4 Department of Computer, Control, and Management Engineering, University of Rome La Sapienza, Rome, Italy; 5 Department of Neurology, University Hospital of Zurich, Zurich, Switzerland; 6 Department of Physical Therapy, University of Applied Sciences Western Switzerland (HES-SO Valais), Leukerbad, Switzerland; 7 Clinic of Plastic Surgery, Padova University Hospital, Padova, Italy; 8 Politecnico di Torino, Turin, Italy; 9 Rehabilitation Center Valens, Valens, Switzerland; 10 University of Geneva, Geneva, Switzerland

Correction to: *Scientific Data* 10.1038/s41597-020-0380-3, published online 10 February 2020

During the typesetting process, multiple errors were introduced into the numbering of the references in this Data Descriptor. These errors have been corrected in the HTML and PDF versions.

